# The ultrasound assessment of osteoarthritis: the current status

**DOI:** 10.1007/s00256-023-04342-3

**Published:** 2023-04-15

**Authors:** Mika T. Nevalainen, Antti-Pekka Uusimaa, Simo Saarakkala

**Affiliations:** 1 Research Unit of Health Sciences and Technology, Faculty of Medicine, University of Oulu, POB 5000, FI-90014 Oulu, Finland; 2 Department of Diagnostic Radiology, Oulu University Hospital, P.O. Box 50, 90029 Oulu, Finland

**Keywords:** Hand, Knee, Osteoarthritis, Ultrasound

## Abstract

Traditionally, osteoarthritis (OA) is diagnosed with the clinical examination supplemented by the conventional radiography (CR). In the research literature, the role of ultrasound (US) imaging in the diagnostics of OA has risen steadily during the last two decades. US imaging is cheap and globally widely available often already in primary healthcare. Here, we reviewed the most essential US literature focusing on OA diagnostics and progression prediction using the various search engines. Starting from the year 2000, our search provided 1 445 journal articles. After reviewing the abstracts, 89 articles were finally included. Most of the reviewed articles focused on the imaging of knee and hand OA, whereas only a minority dealt with the imaging of hip, ankle, midfoot, acromioclavicular, and temporomandibular joints. Overall, during the last 20 years, the use of US imaging for OA assessment has increased in the scientific literature. In knee and hand joints, US imaging has been reported to be a promising tool to evaluate OA changes. Furthermore, the reproducibility of US as well as its association to MRI findings are excellent. Importantly, US seems to even outperform CR in certain aspects, such as detection of osteophytes, joint inflammation, meniscus protrusion, and localized cartilage damage (especially at the medial femoral condyle and sulcus area). Based on the reviewed literature, US can be truly considered as a complementary tool to CR in the clinical setup for OA diagnostics. New technical developments may even enhance the diagnostic value of the US in the future.

## Introduction

Traditionally, osteoarthritis (OA) is diagnosed with the clinical examination supplemented by the conventional radiography (CR). In some cases, magnetic resonance imaging (MRI), available only at specialized healthcare, is used for confirming the clinical OA diagnosis. On the other hand, besides the rheumatology specialty, ultrasound (US) imaging has not been generally used as part of the clinical pipeline for OA diagnostics.

When examining the scientific literature, however, the role of the US in the OA diagnostics has risen steadily during the last two decades. The benefits of US assessment of joints include the evaluation of soft-tissue changes related to OA in addition to the lineation of bony surface contour around the joint. In addition to evaluation of structural OA changes, US offers insight to inflammatory findings thus complementing traditional CR imaging. The major drawback of the US assessment of joints is the lack of visualization within the intra-articular structures.

Another advantage of US imaging is that it is cheap and globally widely available already in several primary healthcare facilities. Consequently, the US can be considered as a complementary tool next to CR. Therefore, US provides an intriguing approach to the modern imaging of the OA, and potentially a new tool in the clinical setup for OA diagnostics.

In this narrative review, we will review the most essential US literature focusing on OA diagnostics and progression prediction. After reviewing the abstracts, 89 articles were included in this review. The majority of the reviewed articles focused on the imaging of knee and hand OA, whereas only a minority dealt with the imaging of hip, ankle, midfoot, acromioclavicular and temporomandibular joints.

## Methods

A literature search was performed with no restrictions on publication type or language within the following databases: PubMed, MEDLINE (via OVID) and The Cochrane Central Register of Controlled Trials, from the earliest records published starting from January 1st of the year 2000. The first search was performed in April 7th 2022, and updated on February 23rd 2023. Search terms covered the following domains (only human studies): sonography/sonographic, ultrasound, ultrasonography/ultrasonographic and osteoarthritis. Reference and citation tracking of included articles and related reviews within the topic was performed to detect further studies.

The articles returned from the search were screened as follows: two reviewers screened titles and abstracts independently against the eligibility criteria in the first stage (roughly 50%-50%). In the second screening round, full-text versions of the potentially relevant studies were screened by the reviewers. When necessary, potentially interesting articles were discussed together. Reasons for exclusion of full-text articles were recorded. The primary eligibility criterion was the use of the US to assess OA. The secondary eligibility criteria were the symptom or pain correlation of the US, assessment of OA progression or comparison of imaging modalities (against US).

Ultimately, the search identified 1 445 papers. No additional studies were identified through previous reviews and citation tracking of included articles. After title and abstract screening and the full-text screening, 89 studies met the eligibility criteria for this review.

### The knee joint

The majority of the literature on US assessment of OA focuses on the knee joint. In general, US is capable of evaluating tibiofemoral osteophytes, effusion / synovitis and meniscus protrusion reliably especially in the medial compartment. Concerning the assessment of articular cartilage, only a limited view of the femoral trochlear and condylar cartilage is reached via US; to date it remains somewhat elusive, whether we are looking at patellofemoral or tibiofemoral joint on this view. To address this issue, Kauppinen et al. (2021) studied 20 healthy knees with US and isotropic 0.6 mm MRI with 90-degree flexed knee to assess the capability of US to visualize the femoral articular cartilage. The authors concluded that up to two-thirds of the articular cartilage of the medial femoral condyle, and one-third in the lateral femoral condyle, can be assessed by US [1].

Only a couple of studies with surgical gold standard exist. Applying knee arthroscopy as the gold standard in a series of 40 patients, Saarakkala et al. (2012) found that association of cartilage changes between US and arthroscopy was significant at the sulcus, at the medial femoral condyle, but insignificant at the lateral condyle; the sensitivities varied between 52–83%, and specificities between 50–100% [2]. Later, using total knee arthroplasty as gold standard, Nevalainen and colleagues (2018) showed in a series of 57 late-stage knee OA patients, that the sensitivity of US was excellent on the medial aspect of knee joint: 92% for medial femoral cartilage damage, 90–95% for medial osteophytes, and 97% for effusion and synovitis [3]. The only histological work was carried out by Lee et al. (2008), who found that with 172 femoral condyles, the in vivo US grading of cartilage was significantly correlated to in vitro US grading and histologic grading [4].

Several studies have examined the US findings as a prognostic tool for knee OA. Ishibashi et al. (2022) studied 404 subjects without radiographic knee OA during three years; At the last follow-up, 114/349 knees (32%) had progressed from non-OA and 32/55 knees (58%) had progressed from early knee OA to radiographic OA. Moreover, female sex, body mass index, and knee effusion were significantly linked with the OA progression [5]. In a 5-year follow-up cohort study of 944 knees, Chiba et al. (2020) demonstrated that subjects with more extruded medial meniscus had a higher prevalence of radiographic OA after the follow-up period. Moreover, by using the threshold 4 mm of meniscal extrusion, the US could identify subjects having a risk of developing OA [6]. However, with a healthy subject cohort (299 participants), Sarmanova et al. (2019) challenged the present cut-offs for effusion (4 mm) and synovial hypertrophy (2 mm) stating that normal ranges for effusion would be 0–10 mm and for synovial hypertrophy 0-7 mm [7]. Indeed, an increase in medial meniscus extrusion may be an important risk factor for the progression of knee OA, and could be feasible to predict the onset of radiographic knee OA in a 5-year follow-up (*n* = 55) [8]. This finding was observed already previous by Kawaguchi and colleagues (2012), since medial meniscus extrusion increased with weight bearing and during 1-year follow-up; furthermore, meniscus extrusion was associated with higher KL grade (*n* = 98) [9]. Furthermore, Bevers and colleagues (2015) described an association between Baker’s cyst and synovial hypertrophy with 125 patients at baseline and radiological and clinical progression in 2-year follow-up [10] (Bevers et al. 2015), and Conaghan et al. (2009) stated that US-detected effusion seems to be a predictor of knee arthroplasty in a 3-year follow-up (*n* = 531) [11].

## Modality comparison

The comparative studies—US against CR, computed tomography (CT) or MRI—generally show rather good diagnostic performance of US. Most recently, Chiba and colleagues (2022) studied 135 knees with and without OA changes demonstrating that on US greater medial meniscus protrusion was associated with higher medial meniscus root tear seen on MRI; the authors suggested 5 mm and 7 mm cuf-offs of US medial meniscus protrusion for non-OA and OA cases, respectively [12]. Abicalaf et al. (2021) reported that the higher KL grades were correlated to a higher number of US findings especially when comparing patients of lower and higher grades—i.e. KL I and IV, KL I and III, KL II and IV) [13]. Moreover, Jiang et al. (2021) reported that in a Chinese population of 3755 participants (mean age 64.4 years), the synovial abnormalities were associated with radiographic knee OA [14].

**Fig. 1:**
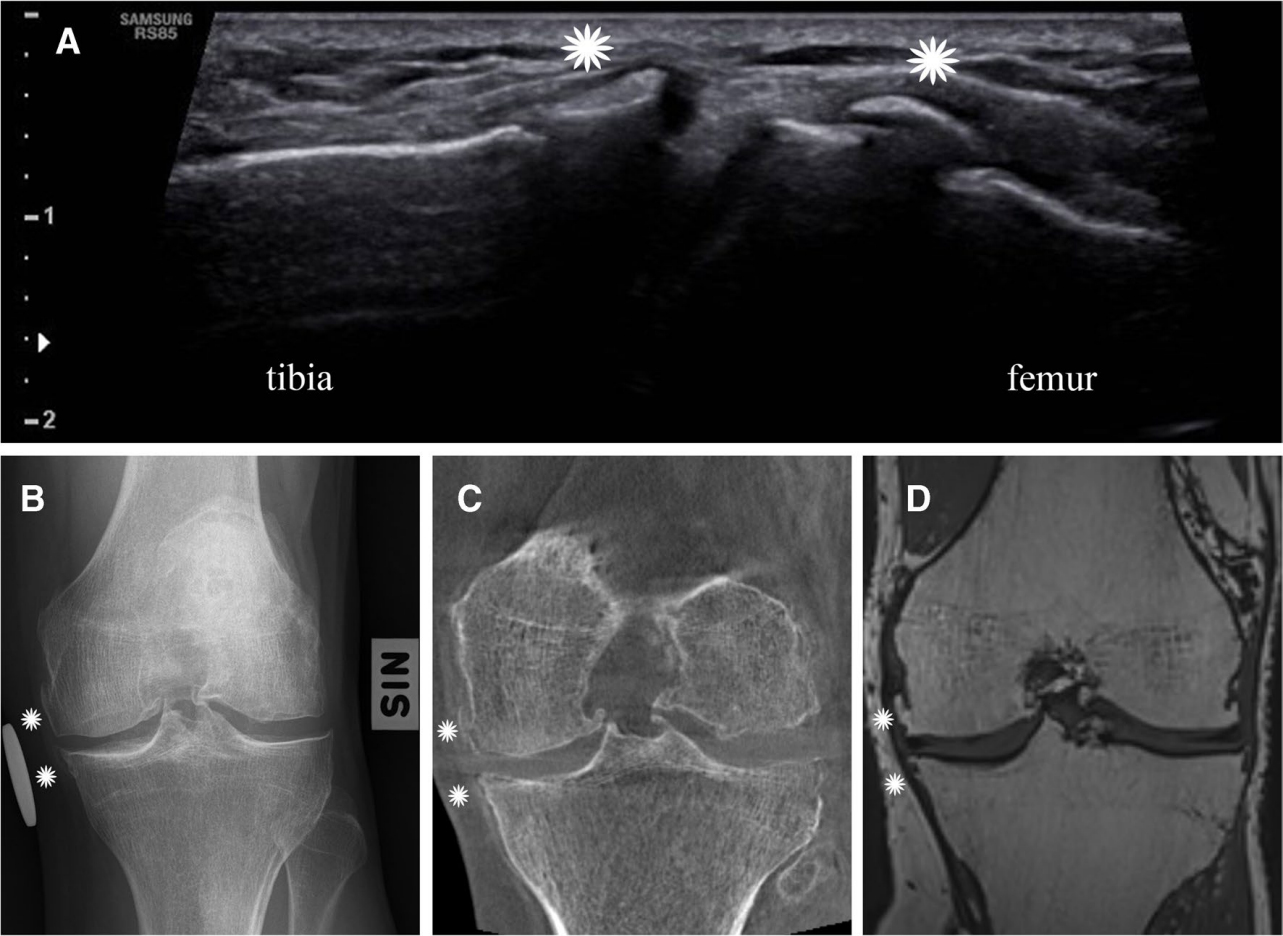
A 54-year-old male with medial osteophytes (white asterisks) of the knee joint as depicted by longitudinal US (**A**), anteroposterior CR (**B**), coronal CT (**C**), and coronal T1-weighted MRI plane (**D**). In general, all the modalities provide good visualization of the osteophytes (white asterisks). However, based on the multiple studies reviewed here, the US seems to be superior to CR for detection of osteophytes

Oo et al. (2022) demonstrated that the correlations between quantitative US pathologies and corresponding MRI findings were very strong (ICC range = 0.85–0.98) excluding lateral meniscal extrusion (ICC 0.66) [15]. The same group assessed 89 knee OA patients to find out if US findings are associated with CR and MRI findings. All US scores (excluding PD) were significantly correlated with KL grade. Moreover, US findings (excluding cartilage damage) demonstrated at least moderate correlation with their MOAKS counterparts [16]. de Vries et al. (2020) studied 31 knee OA patients concluding that routine grayscale US has limited overall accuracy for detecting synovitis when using gadolinium enhanced MRI as the gold standard. When US is combined with Power Doppler or contrast-enhanced US, the diagnostic performance improves for detecting mild synovial inflammation [17]. With 57 late-stage OA patients, Nevalainen and collegues showed that the US detection rate of femoral cartilage damage was in line with the radiographic joint space narrowing. Concerning the osteophytes, US provided superior results to radiography particularly on the medial side [3]; same results were achieved a few years earlier by Koski et al. (2016), who showed that US detected more osteophytes than CR at the medial (65% vs. 48%) and lateral (70% vs. 60%) compartments [18]. Podlipska et al. (2016) compared the diagnostic performance of US and CR against MRI in 79 OA and 80 healthy subjects; US performed best in the assessment of femoral medial and lateral osteophytes, and medial meniscal extrusion. In comparison to radiography, US was superior or at least equally good in identification of tibiofemoral osteophytes, medial meniscal extrusion and medial femoral cartilage changes [19]. In addition, positive correlation with KL grades has been shown with osteophytes and cartilage damage at US assessment [20, 21], but also with effusion and synovial hypertrophy [22]. Okano and colleagues (2016) reported a strong correlation between the radiographic medial tibiofemoral narrowing and the US medial cartilage damage, and between medial and lateral osteophytes in both modalities. Furthermore, the US detected marginal osteophytes when CR did not detect any osteophytes with 166 knees [23]. Last, Abraham and colleagues (2014) studied 311 subjects aged 61–63 years with US examination of knee, concluding that US is more sensitive than CR to detect OA, especially osteophytes [24]. Figure 1 shows an example how osteophytes are depicted on US and other imaging modalities.

## Association with pain and symptoms

Although the overall association between the imaging findings and pain or symptoms related to OA is traditionally known to be rather poor, the recent US literature suggests otherwise. Philpott et al. (2022) studied the association between US-detected synovitis (both grayscale and Power Doppler) and pain in 248 knee OA patients (in total 453 knees) concluding that moderate or severe synovitis was strongly associated with constant pain. In the subgroup analyses of the radiographic early-stage OA (KL ≤ 2), the synovitis was linked with both the intermittent and constant pain, whereas in the radiographic late-stage OA (KL ≥ 3) subgroup pain association was absent [25]. In the same year, Saito et al. (2022) examined 1 667 subjects aged over 60 years reporting that medial meniscus protrusion and medial osteophytes, age, and body mass index showed significant associations with KSS symptom scores. They concluded that US findings were more related to KSS symptoms than radiographic KL grades [26]. With 245 patients suffering from knee OA, Mortada and colleagues (2022) used US sum score to show that association between WOMAC subscales (pain, stiffness and function) and VAS exists—moreover, the US sum score was able to categorize patients groups with no or mild pain, moderate pain, and severe pain [27]. Abicalaf et al. (2021) studied 194 OA knees showing a significant moderate positive association between VAS scores and the number of US findings. Moreover, there was a significant association between the number of US findings and WOMAC scores for pain and physical function domain [13]. In a study of 89 knee OA patients Oo and colleagues found out that synovial hypertrophy, power Doppler and meniscal extrusion were associated with higher pain. Furthermore, all US scores, excluding cartilage damage, showed significant associations with worse KOOS symptoms [16]. Similarly, in a Chinese population of 3755 participants (mean age 64.4 years), the synovial abnormalities were associated with knee pain [14].

Also studies conducted before the 2020s show some associations between US findings and OA-related pain or symptoms. Kandemirli et al. (2019) showed an association between the Baker’s cyst and VAS and WOMAC scores with 99 patients [28], and Kauppinen et al. (2020) depicted a strong association between medial-sided pain and same-sided cartilage damage and osteophytes, but concluded that US findings show only a poor association with clinical OA findings according to KSS with a study of 123 OA knees [29]. With 113 knee OA subjects, Bernardo-Bueno et al. (2019) demonstrated that structural OA changes—including cartilage abnormalities, medial meniscus protrusion and medial femoral osteophytes—were able to predict severe pain in knee OA; however, inflammatory findings did not contribute to joint pain [30]. Previously, several authors have observed association between the US-detected OA findings and WOMAC scores (both pain and physical function) [20, 31–35]. Moreover, especially the inflammatory findings—namely effusion and synovial hypertrophy—have been reported to associate with increased knee pain in OA [20, 36–38].

## Reproducibility

The overall reliability of the US to assess knee OA has been found good to excellent over the years, and both intra- and inter-rater agreement has been evaluated in multiple studies. In the most recent study involving 89 subjects, Oo et al. (2022) showed excellent inter-rater agreement on quantitative US evaluation for osteophytes (ICC range = 0.90–0.96), meniscal extrusion (ICC range = 0.90–0.93), and synovitis (ICC range = 0.86–0.88) [15]. Same results were achieved by Kauppinen et al. (2020), who reported that the inter-rater agreement was excellent on the medial side of the knee joint (PABAK = 0.811–0.887) [29], and by Razek et al. (2016), who found an excellent inter-rater agreement (k = 0.86–1.00) with 80 knee OA patients [33]. Furthermore, three studies including several sonographers have confirmed the good reproducibility of the knee US: Between 11 experienced sonographers, intra- and inter-rater agreements were moderate to good for the global synovitis (0.70 and 0.50, respectively), and the mean intra- and inter-rater agreement for cartilage damage, medial meniscal pathology and osteophytes ranged from fair to good (0.55 and 0.34, 0.75 and 0.56, 0.73 and 0.60, respectively) [39]. Koski et al. (2016) evaluated the intra- and inter-rater agreement on osteophytes with 40 patients and 14 readers with US observing that intra- and inter-rater US agreement was very good (κ = 0.60–0.72) [18]. Iagnocco et al. (2012) studies 17 knees for inter-rater agreement with US reporting that inflammatory abnormalities were detected with moderate to very good agreement (k = 0.55—0.88). From fair to very good agreement (k = 0.31—0.82) was seen between sonographers for structural lesions. The overall k was 0.716 for junior and 0.571 for beginner sonographers as compared to senior sonographers [40].

## Recent technical developments

Besides the conventional 2D US imaging using grayscale and Power Doppler, we found a few interesting studies aiming to produce technical and methodological advances to clinical US imaging of joints in the future. Most recently, Zhang et al. (2022) studied 60 patients with early knee OA to assess the feasibility of shear wave elastography to diagnose OA. The authors found a strong negative correlation between shear wave speed of the quadriceps tendon and the degree of OA, proposing that elastography could be used in the evaluation of knee OA [41]. Another study using shear wave elastography was conducted by Yokus et al. (2020), where the authors studied 40 OA knees and 40 healthy knees with routine US and shear wave elastography concluding that cartilage thickness measurements were similar between the two groups, but the shear wave velocity values in the cartilage were significantly higher in the OA group [42]. Regarding novel Doppler techniques, Oo et al. (2019) compared superb microvascular Doppler to conventional Power Doppler imaging in 89 patients to detect knee synovitis using MRI as reference standard. The authors concluded that superb microvascular Doppler is superior to detect low-grade inflammation in knee OA, and can reveal significant associations with symptoms, CR findings and MRI-detected synovitis. However, the added clinical value of this technique remained unclear [43].

Regarding the 3D US imaging which is nowadays possible with the modern US equipment, Vendries et al. (2021) compared 3D US with CT to detect osteophytes in three cadaver knees. The authors did not find statistical difference between the modalities, which led to the conclusion that 3D US imaging can improve the detection of osteophytes with cartilaginous components [44]. Finally, regarding the proper positioning of the knee joint during US imaging, Sorriento and colleagues (2021) developed a wearable knee brace to standardize the US acquisition. They recruited three untrained subjects who used the brace to successfully self-acquire US images [45].

### The hand joints

Traditionally, hand OA has been studied much less than knee OA. However, in the recent decade, we have seen an increased interest also for hand OA. When considering the capability of US imaging to predict hand OA progression, in the landmark study by Mathiessen et al. (2016) authors reported that US grayscale synovitis and Power Doppler signals were clearly associated with radiographic progression of hand OA within a 5-year follow-up in a cohort of 78 subjects [46]. Regarding erosive hand OA, Kortekaas et al. (2016) studied the association between US inflammation and development of erosive disease diagnosed by CR within 2-year follow-up with 56 hand OA patients. They found that US-detected inflammatory findings are associated with the development of erosions [47]. Furthermore, with the same patient cohort, Kortekaas et al. (2015) assessed 1680 joints with US and CR, showing associations between synovial thickening,effusion and Power Doppler signal, and progression of osteophytes and of joint space narrowing [48]. Finally, Damman et al. (2016) found positive associations between Power Doppler signal and synovial thickening at baseline and progression of joint space narrowing in 2-year follow-up [49].

In the cross-sectional study design, Kortekaas et al. (2013) evaluated 18 IP joints from 55 hand OA patients with US and CR concluding that inflammatory US findings are more common in erosive OA despite the number of erosive joints in general [50]. Furthermore, Arrestier et al. (2010) examined 880 IP joints of hand OA patients and 736 IP joints of healthy controls, observing that the DIP effusion was significantly higher in the patients. Increased Doppler signal was rare [51].

## Association with pain and symptoms

In the hand OA, multiple studies have been published during the last decade focusing on the association between US-detected features from the hand joints and symptoms, primarily pain. Most recently, Shi et al. (2022) studied 166 subjects with symptomatic CMC I joint OA showing a significant association between US Power Doppler signal and patient's global assessment (PGA). The radiographic joint space narrowing was associated with the changes in stiffness and PGA from baseline to the 12-week follow-up. The authors found no association between US findings and changes in the clinical outcomes within 12 weeks [52]. In contrast, in a small cohort of 33 symptomatic hand OA subjects and 26 healthy controls, no association was found between VAS pain and MRI/US findings [53].

In 2020, with 285 subjects with hand OA, Steen Pettersen and colleagues (2020) reported that the severity of structural pathology and hand joint inflammation was associated with lower pressure pain thresholds in the finger joints, suggesting that pain sensitization could be driven by structural and inflammatory changes in hand OA [54]. Furthermore, Fjellstad et al. (2020) examined 290 hand OA patients to study whether gray-scale of Power Doppler synovitis are associated with pain and physical function. Bilateral interphalangeal and CMC1 joints were scanned with US, and inflammation in both regions was associated with pain in the joints. However, associations with hand pain, reduced physical function, and lower grip strength were only detected for inflammation in the CMC I joints [55]. Still in the same year, Oo et al. (2019) published a study where they examined 93 subjects with CMC I joint OA depicting that Power Doppler signal has a significant association with VAS pain. US osteophyte grade was significantly associated with radiographic KL grade and OARSI osteophyte & joint space narrowing grades. There was also an association between US and CR-detected erosions [56].

In 2017, Spolidoro Paschoal and colleagues (2017) performed a prospective double-blind study of 60 interphalangeal joints examining grayscale synovial hypertrophy and Power Doppler signal against clinical and functional findings. The joints were assessed six times per year by clinician and sonographers. The authors concluded that joint swelling, grip and pinch strength, and the AUSCAN Index were weakly associated with US findings in proximal interphalangeal joints [57]. In contrast, Kroon and colleagues (2018) studied CMC I and STT joints with MRI, US and CR (*n* = 87). They observed no association between inflammatory US findings and pain [58].

Earlier, Mallinson et al. (2013) studied 31 patients with symptomatic OA and 37 asymptomatic controls with US of CMC I joint. Only osteophytes were associated with patient disability. However, all US findings demonstrated statistically significant higher grades in the symptomatic group [59]. Furthermore, with 55 patients, in total of 1649 hand joints, Kortekaas et al. (2011) evaluated the associations between structural US or CR findings and pain. They concluded that on US 69% showed osteophytes and on CR 46%, joint space narrowing was seen in 47%. Moreover, osteophytes and JSN displayed independent associations with pain [60]. Finally, Kortekaas et al. (2010) evaluated 55 hand OA patients with the US-detected grayscale synovitis, effusion and Power Doppler signal associating with pain (AUSCAN) [61].

## Modality comparison

There are several studies published where the US has been compared with other imaging modalities or histology. In most of the studies, US has been compared with CR or MRI, but there is a very recent study by Husic et al. (2022) where the structural changes between US, micro-CT (mCT) and histology in subjects suffering from hand OA in 31 fingers were compared. Authors analyzed 992 regions with US, micro-CT and histology, stating that a comparable number of erosions was detected with each method. Both imaging techniques correlated moderately with each other regarding the detection of osteophytes (r = 0.54) and erosions (r = 0.43). However, the correlation of US or mCT with histology concerning erosions or osteophytes was poor. When using histology as the gold standard, US had a sensitivity of 80% and a specificity of 32% to detect osteophytes, whereas the respective numbers for micro-CT were 73% and 27%. For erosions, sensitivities (US 10% and mCT 6%, respectively) were significantly lower [62].

In another recent study, Eymard and colleagues (2022) assessed 33 symptomatic hand OA subjects and 26 healthy controls with MRI and US. They found that the prevalence and severity of most lesions detected by MRI or US were higher in OA patients. In that study, excluding osteophytes, MRI seemed to be more sensitive than US. Perhaps the most important finding in the study was that US was both sensitive (91%) and specific (93%) for detecting osteophytes in joints without radiographic findings [47]. Furthermore, Sivakumaran et al. (2018) reported that erosions and osteophytes detected with US are significantly associated with corresponding CR findings. They suggested that US is more sensitive for diagnosing hand OA than CR [63]; later Güven et al. (2020) have reached the same conclusion (Güven et al. 2020). These findings agree with the ones reported already in 2013, when Mathiessen et al. studied the reliability of US evaluation of osteophytes and the intermodality concordance by US, CR, MRI, and clinical joint examination in 127 patients with hand OA. US had 83% sensitivity and 75% specificity to detect osteophytes compared with MRI. US found moderate or large osteophytes more often than MRI, detected more osteophytes than CR or clinical examination [64].

Besides osteophytes, US has been also reported to significantly associate with other MRI findings. Vlychou et al. (2013) evaluated 240 joints (MCP and IP joints) both by US and MRI. They reported excellent agreement between the modalities for cysts (κ = 0.85), erosions (κ = 0.84), synovitis (κ = 0.82) and tenosynovitis (κ = 0.83), and substantial agreement for osteophytes (κ = 0.79) [65]. Furthermore, Wittoek et al. (2010) reported high agreement between US and MRI for erosions, osteophytes and synovitis, and they also showed better performance of US for detecting interphalangeal erosion compared to CR [66]. Finally, already in the year 2007 Keen et al. (2008) found that US detected more osteophytes and joint space narrowing than CR in 1106 joints of 37 hand OA patients [67].

## Reproducibility

Similarly as with knee OA, the overall reliability of the US to assess hand OA has been found good over the years, and multiple intra- and inter-rater agreement studies have been published after the year 2000. Most recently, Mathiessen and colleagues reported that the web-based reliability exercise with 99 static US images showed good intra- and interreader agreement for all inflammatory features (κ > 0.64). In the patient-based exercise (six sonographers, 12 hand OA patients), intra- and interreader agreement varied more (κ = 0.45—0.90), but the percentage close agreement was high for all US findings (> 85%) [68]. Similarly, van de Stadt et al. (2022) studied the agreement between real-time and static US in 75 hand OA patients (2250 joints) stating that agreement was good to excellent at the joint level (k 0.72–0.88) and moderate to excellent at the patient level (ICC 0.58–0.91). Importantly, they also concluded that real-time scoring of US images should remain the standard for clinical hand OA trials [69].

In 2020, Oo et al. (2020) examined 40 subjects with CMC I joint OA using US reporting a intra-rater agreement of 0.81 (PABAK) [56]. Spolidoro Paschoal and colleagues (2017) performed a prospective double-blind study of 60 interphalangeal joints examining grayscale synovial hypertrophy and Power Doppler signal against clinical and functional findings. They reported good intra- and inter-rater agreement for the US assessments (ICC 0.474–0.857; k 0.390–0.673) [57]. Damman et al. (2016) assessed 56 hand OA patients concluding that US reliability was from intermediate to good (ICC for PDS 0.62, synovial thickening 0.93) [49]. With 10 hand OA patients and 10 experienced sonographers, Hammer et al. (2014) assessed the reliability of US to detect osteophytes and cartilage damage concluding that the intra- and inter-rater agreements were substantial to excellent (k = 0.68–0.89) for osteophytes, and fair to moderate (k = 0.46–0.66) for cartilage damage [70]. Usón et al. (2014) examined 50 painful and 50 non-painful interphalangeal joints with US and CR. The symptomatic joints showed significantly more osteophytes, synovitis and cartilage damage on US compared to CR, and intra-rater US agreement was excellent (up to 98–100%, without reported statistics, though) [71].

In older studies published more than 10 years ago, intra- and inter-rater reliability of US has been also generally at the good level. Mathiessen et al. (2012) reported excellent intra-rater (0.95–0.98) and inter-rater (0.91–0.96) reliability (*N* = 127) for detecting osteophytes [64]. In erosive hand OA, Wittoek et al. (2011) reported substantial inter-rater agreement for erosions, osteophytes, synovitis and effusion in IP joints (*n* = 252; k > 0.80) [66]. Same authors also reported one year earlier excellent inter-rater agreement for several US-detected features in IP joints (*N* = 38; κ = 0.91 for erosions, κ = 0.98 for osteophytes, κ = 0.98 for effusion, κ = 0.99 for greyscale synovttis, κ = 0.94 for Power Doppler signal [72]. In 2009, Mancarella et al. (2009) reported an intra-rater agreement of 0.86–0.94 (*n* = 700) for synovial hypertrophy, effusion and Power Doppler signal [73]. Keen et al. (2008) reported an intra-rater agreement of k = 0.83 for osteophytes and 0.64 for joints space narrowing (*N* = 37; *n* = 1106) [67]. Finally, regarding the US-evaluated cartilage damage of MCP joints, Iagnocco et al. (2012) reported intra-rater agreement of 0.52–1.00 and inter-rater agreement of 0.80 with 32 joints [74].

### Other joints

#### Hip

**Fig. 2:**
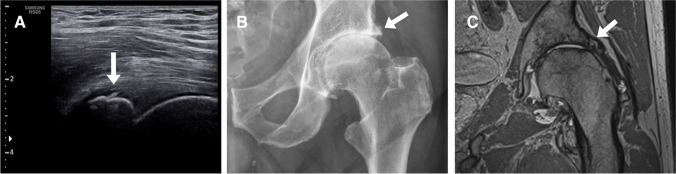
A 68-year old female with a large acetabular osteophyte (white arrow) visualized in longitudinal US (**A**), anteroposterior CR (**B**), and coronal PD-weighted MRI plane (**C**). The osteophyte is nicely delineated in each modality. Similarly as in the knee joint, the literature supports that US is at least comparable to CR for detection of hip joint osteophytes. Moreover, for the femoral head deformity, the diagnostic performance of the US was even superior to CR [68]

To date, only a few studies on US evaluation of the hip joint exist. Primary reason explaining this fact is that the deep location of the hip joint creates major challenges to the US assessment lowering its diagnostic value to detect OA changes. Using the hip arthroplasty as gold standard, Nevalainen et al. (2020) studied 48 late-stage hip OA patients showing a 95% sensitivity for femoral collum ostephytes, 96% sensitivity for acetabular osteophytes, 92% sensitivity for femoral head deformity, and 49% sensitivity for effusion; however, the specificities and accuracies remained rather low. Interestingly, US showed a similar detection rate of osteophytes as CR, and for the femoral head deformity, the diagnostic performance of the US was superior to CR [75]. Figure 2 shows an example of an acetabular osteophyte detected on US, CR and MRI. Concerning disease progression, only one study by Birn and colleagues (2013) exists; the authors studied 94 hips with US and concluded that large joint effusions are associated with radiographic findings of rapidly destructive osteoarthritis [76]. Moreover, the association between US-detected effusion and joint aspiration has been shown to be surprisingly poor. With 100 hip OA patients, no association between aspiration and fluid on US was detected confirming the challenges on hip joint assessment on US [77].

Concerning the pain or symptom association and reproducibility, the results vary to some extent. With 66 patients, the inter-rater agreement of the US evaluation varies from moderate to excellent (k = 0.54–0.82), and no association between US and OHS was detected [75]. Clausen et al. (2020) studied 92 hips with OA reporting excellent inter-rater reliability for hip effusion/synovitis (k = 0.90) and general osteoarthritis grading (k = 0.80). More spesifically, for acetabular and femoral osteophytes, femoral cartilage defects, femoral head deformity, and labrum changes κ-values were lower ranging from 0.39 to 0.70. Concerning the intrarater reliability (based on the still US images reviewing), equal or superior reliability was reached (k = 0.70–0.87). However, the agreement between US and CR findings (*n* = 63) was rather poor (κ = 0.21—0.46) [78]. Using a cohort of 107 subjects, Kemp et al. (2020) found no association between US measured hip effusion, HOOS score and CAM morphology [79]. With 150 OA hips evaluated by US, Iagnocco et al. (2012) reported that the hip pain associated significantly with the effusion; moreover, age and disease duration was associated with the osteophytes. However, the US-detected abnormalities were present also in asymptomatic patients [80]. Qvistgaard et al. (2006) examined 100 hips with OA to determine intra-rater and inter-rater agreement of hip US: The intra-rater agreement showed good to excellent correlation, 0.8 for osteophytes, 0.78 for femoral head deformity, 0.71 for effusion, and 0.69 for synovitis. The Inter-rater agreement was only fair to good with corresponding values of 0.65, 0.63, 0.45 and 0.6, respectively. The authors also found an association between the pain on VAS and the US scores for global osteoarthritis and osteophytes [77].

#### Ankle

As the ankle joint is not very suitable for US assessment, only one study on ankle OA was found in the literature. Nevalainen et al. (2021) studied 51 patients suffering from ankle OA with CR, US and conebeam-CT; they found that US detected effusion/synovitis within the talocrural joint with 45% sensitivity and 90% specificity. Concerning the anterior talocrural osteophytes, US had a sensitivity of 78% and specificity of 79%. For the medial osteophytes, they were 39 and 83%, and for the lateral osteophytes 54 and 100%, respectively. For the cartilage damage of the talus, US showed a low 18% sensitivity and a high 97% specificity. Taken together, the performance of US was only moderate, but comparable to CR. The imaging findings showed only weak associations with ankle symptoms according to WOMAC [81].

#### Midfoot

Concerning midfoot, three studies were identified: Zabotti et al. (2019) sought out to evaluate the reproducibility of the midfoot US for assessing synovial hypertrophy, effusion, cartilage damage, osteophytes and Power Doppler score by 11 rheumatologists. Using 110 static US images, the intra-rater agreement was 0.49–0.90 (PABAK) and inter-rater agreement 0.60–0.89. With real-time US evaluation of 12 patients, the intra-rater agreement was 0.62–0.95, and inter-rater agreement 0.36–0.93. The authors concluded that standardized US definitions were reliable for assessing inflammatory lesions, whereas the evaluation of structural damage needs further studies [82]. Camerer and colleagues (2017) assessed 2445 midfoot joints in 124 patients, reporting that US detected more osteophytes than CR (344; 14.1% vs.13; 0.5%), and more erosions (60; 2.5% vs. 3; 0.1%). The authors stated only a weak agreement between the two modalities (κ 0.029–0.035) [83]. With 200 osteoarthritic feet, Iagnocco et al. (2011) reported inflammatory abnormalities in 87/200 midfoot (43.5%) and structural lesions in 100/200 (50%) midfoot. At forefoot, US detected inflammatory abnormalities in at least one joint in 176 feet (88%) and structural pathologies in 189 feet (86%) [84].

#### AC joint

The studies examining the AC joint are also sparse. With a study group of 212 shoulders (patients aged 50–75 years), Khoschnau et al. (2020) found no association between acromioclavicular degeneration—on US and CR—and shoulder complaints or Constant score [85]. In a recent review, Precerutti and colleagues (2020) stated that due to the vast number of US examinations performed to investigate shoulder pain, the AC joint should be included in the routine assessment. The authors conclude that the US can easily detect AC osteoarthritis [86]. With 51 asymptomatic male subjects aged 40–70 years, Girish et al. (2011) found the prevalence of AC joint OA 65% on US examination [87]. In a rather old study, Blankstein et al. (2005) examined 30 adult subjects with shoulder pain and 30 asymptomatic controls to evaluate US on AC joint pathologies; effusion, osteophytes, joint space narrowing/widening, cysts and calcifications were found, all these findings representing early degenerative findings of OA. The conclusion was that US should be used as complementary modality in addition to routine CR evaluation [88].

#### Temporomandibular joint

We found only one study examining the OA findings of the temporomandibular joint using US. Kothari et al. (2016) performed the US evaluation of synovial thickness and Power Doppler signal by two experienced radiologists in 58 temporomandibular joint pain patients and 41 controls. The US had sensitivity of 60% and specificity of 63% for the diagnosis of OA when clinical diagnosis and CBCT were used as the gold standard [89].

## Conclusions

During the last twenty years, the use of US imaging OA assessment has constantly increased in the scientific literature. The most commonly studied joints are knee and hand, where the US evaluation has been shown to be a promising tool to evaluate OA changes. Furthermore, the reproducibility of US as well as its association to MRI findings are very good. Importantly, US seems to even outperform CR in certain aspects, such as detection of osteophytes, joint inflammation, meniscus protrusion, and localized cartilage damage (especially at the medial femoral condyle and sulcus area in the knee joint).

To summarize, US is an accessible and inexpensive imaging modality providing an enticing tool to assess both the structural and inflammatory changes in osteoarthritic joints. Based on the reviewed literature, US can be truly considered as a complementary tool to CR in the clinical setup for OA diagnostics. New technical developments may even enhance the diagnostic value of the US in the future.
